# ATP and Its Metabolite Adenosine as Regulators of Dendritic Cell Activity

**DOI:** 10.3389/fimmu.2018.02581

**Published:** 2018-11-09

**Authors:** Cinthia Silva-Vilches, Sabine Ring, Karsten Mahnke

**Affiliations:** Department of Dermatology, Ruprecht-Karls-University Heidelberg, University Hospital, Heidelberg, Germany

**Keywords:** dendritic cells, adenosine, CD73, tolerance, ATP

## Abstract

Adenosine (Ado) is a well-studied neurotransmitter, but it also exerts profound immune regulatory functions. Ado can (i) actively be released by various cells into the tissue environment and can (ii) be produced through the degradation of extracellular ATP by the concerted action of CD39 and CD73. In this sequence of events, the ectoenzyme CD39 degrades ATP into ADP and AMP, respectively, and CD73 catalyzes the last step leading to the production of Ado. Extracellular ATP acts as a “danger” signal and stimulates immune responses, i.e. by inflammasome activation. Its degradation product Ado on the other hand acts rather anti-inflammatory, as it down regulates functions of dendritic cells (DCs) and dampens T cell activation and cytokine secretion. Thus, the balance of proinflammatory ATP and anti-inflammatory Ado that is regulated by CD39^+^/CD73^+^ immune cells, is important for decision making on whether tolerance or immunity ensues. DCs express both ectoenzymes, enabling them to produce Ado from extracellular ATP by activity of CD73 and CD39 and thus allow dampening of the proinflammatory activity of adjacent leukocytes in the tissue. On the other hand, as most DCs express at least one out of four so far known Ado receptors (AdoR), DC derived Ado can also act back onto the DCs in an autocrine manner. This leads to suppression of DC functions that are normally involved in stimulating immune responses. Moreover, ATP and Ado production thereof acts as “find me” signal that guides cellular interactions of leukocytes during immune responses. In this review we will state the means by which Ado producing DCs are able to suppress immune responses and how extracellular Ado conditions DCs for their tolerizing properties.

## Adenosine triphosphate (ATP) in peripheral tissues

**Figure 1:**
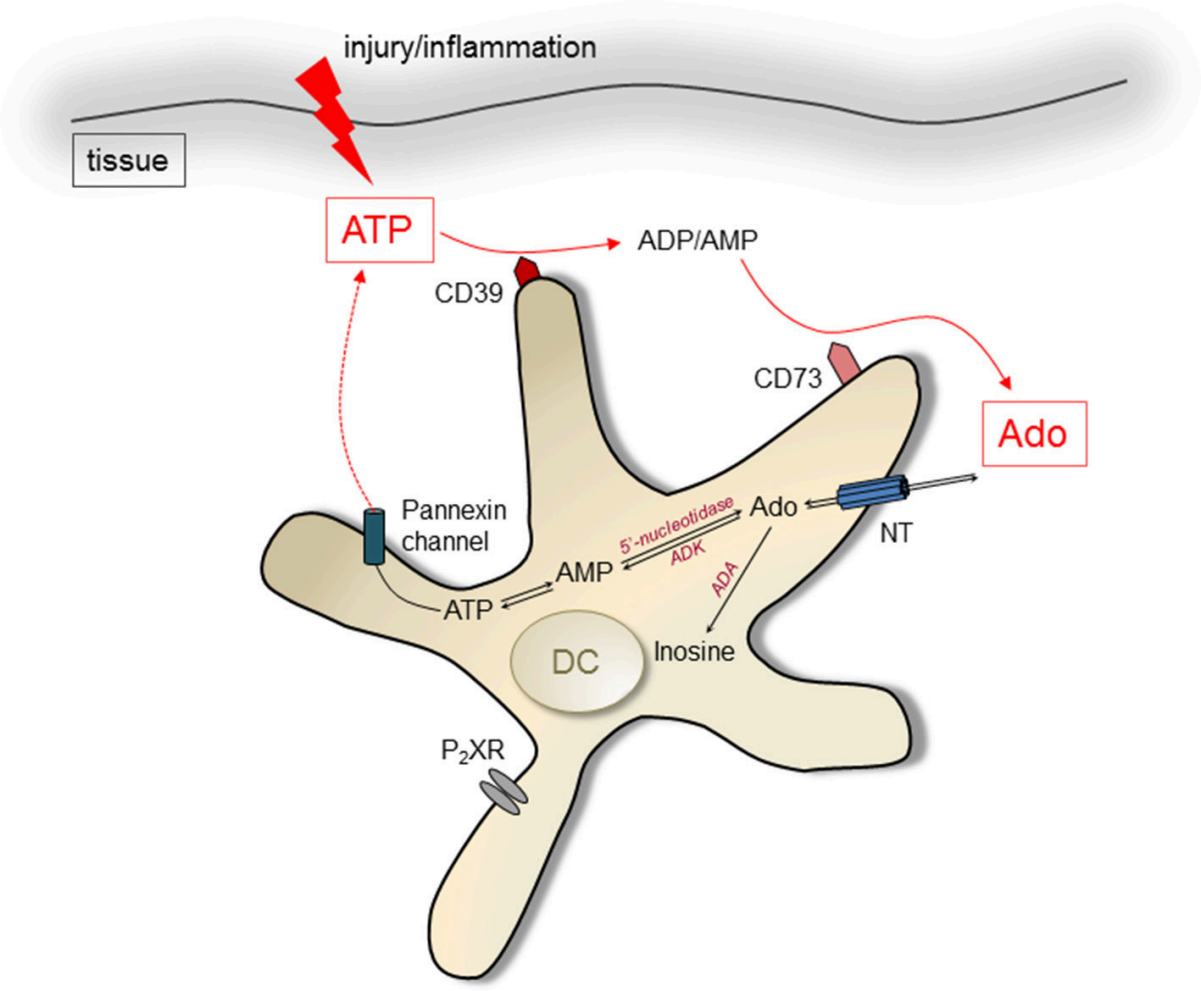
Pathways of ATP/Ado generation in DCs. Intracellular Ado can be produced by degradation of AMP by 5′ectonucleotidases. Nucleoside transporters (NT) lead to extrusion of Ado. ATP can be released by cells via pannexin channels after injury and during inflammation, acting immune stimulatory by engagement of P2X receptors (P2XR). It can be degraded by the ectoenzymes CD39 and CD73, resulting in increased levels of Ado in the extracellular environment. Ado can be degraded by action of the enzyme Adenosine deaminase (ADA) intra- and extracellularly.

The chemical family of purines comprises of heterocyclic aromatic organic compounds, consisting of a pyrimidine ring fused to an imidazole ring. It comprehends biologically active molecules such as Adenosine-triphosphate (ATP) and its degradation product adenosine (Ado). ATP is widely known as an energy carrier within cells, but it can also be released from cells into the environment by cell membrane channels (gap junctions, pannexin channels) or specialized transporters (Figure 1) (1–4). Once located in the intercellular space, ATP transmits signals to other cells by engaging P2 receptors. P2 receptors can be divided into P2X and P2Y subtypes, which comprise different members as indicated by numbers, e.g., P2X_1_ to P2X_7_ and P2Y_1_, P2Y_2_, P2Y_11_. While all P2X receptors bind ATP, only the P2Y_1_, P2Y_2_, and P2Y_11_ receptors are engaged by ATP. The mode of action of P2X and P2Y receptors differs also and can be described as ionotropic for P2X receptors, or metabotropic G-protein coupled in case of P2Y types. The P2X_7_ receptor is a well-studied example and serves as prototypic ATP receptor in many investigations. P2X receptors often form multimeric complexes that upon engagement open a pore for cations such as Na^+^, Ca^2+^, or K^+^ (5). This ion flux will then induce further intracellular signaling events. The most important pathway triggered by P2X receptors involves activation of the NLRP3 inflammasome, leading to caspase-1 activation, which in turn activates interleukin (IL-) 1β and IL-18, two important pro inflammatory cytokines. But this is only one well studied example. In particular the transmembrane flux of Ca^2+^ ions can trigger multiple signaling events in cells involving mitogen activated kinases (MAPK), protein kinase C (PKC) and calmodulin. Therefore, many more effects of ATP induced signaling in leukocytes have been described These comprise the activation of T cells, (6–8), the release of IL-6, TNF (9, 10), prostaglandin (11), CXCL8, CCL2, CCL3 (12, 13) and metalloproteinase 9 (14), just to name a few [comprehensive list in Zimmermann. (15)]. The P2Y_1_ receptor, which is binds ATP in rodents and the P2Y_2_ receptor act via Gq coupled receptors and phospholipase C. Downstream, the second messengers inositol 1,4,5-triphosphate (IP3) that signals further via intracellular Ca^2+^ levels and diacylglycerol (DAC), which activates PKC, are produced. This rather general activation scheme illustrates the diverse groups of effects that can be induced by P2Y receptor engagement. Indeed, involvement of P2Y receptors in regulating hormone release and CNS activity has been documented in many instances. Beyond that, P2Y receptors are expressed by neutrophils, monocytes and T cells, indicating a role for immune regulation as well.

Due to the potent immune stimulatory actions of ATP, the extracellular concentrations are kept in check by enzymatic digestion of ATP. ATP is degraded fast within tissues, making it difficult to investigate its controlled release in defined organs *in vivo*. However, as skin is assessable for manipulation and measurement of ATP (16) and harbors several phenotypically distinct DC subtypes (17), it may be an organ of choice for investigating purine mediated signaling *in vivo*. At first, under non-inflammatory conditions the initial differentiation of skin keratinocytes (KCs) is guided by ATP. Upon binding of ATP the intracellular calcium levels rise gradually (as KCs express different subsets of ATP-specific P2X receptors depending on the layer), inducing the differentiation of the KCs (18, 19). Even the terminal differentiation and subsequent apoptosis of KC in the junction between stratum granulosum and stratum corneum seems to be dependent on ATP. Here, extensive colocalization of P2X_7_ receptors with caspase-3 is evident (20), suggesting induction of cell death by ATP. This is corroborated by *in vitro* data, showing that prolonged engagement of P2X_7_ receptors leads to extended pore-opening enabling even macromolecules of up to 900 Da to travel into cells, leading to induction of caspase-dependent cell death (21). Beyond serving as messenger involved in skin differentiation, ATP has also clear functions as a danger molecule. Due to its function as activator of the NLRP3 inflammasome, ATP is involved in triggering skin allograft rejection. Here it has been shown that ATP is released by host cells in response to transplantation leading to IL-18 production and Th1 responses. Moreover, the skin may “use” ATP even to alert the peripheral immune system, as monocytes during acute rejection of transplants exhibited higher expression of P2X_7_ receptors (22). Skin, as opposed to most other organs, is exposed to UV irradiation. This causes DNA damage, which produces a special set of danger signals. In response to UV irradiation, ATP is released by KCs triggering activation and release of IL-17 by dendritic epidermal γδ T cells (23). Once activated, γδ T cells can release ATP by themselves, leading to an autocrine activation loop maintained by P2X_4_ receptors (24). Functionally this sustained production of IL-17 is of importance for limiting adverse effects of UV, as it upregulates genes necessary for DNA-damage repair, such as TNF-related weak inducer of apoptosis (TWEAK) and the growth arrest gene GADD45 (23). Therefore, in case of UV induced cancers, therapeutic enhancement of extracellular ATP may offer a way for treatment.

Also in chronically diseased skin the distribution of ATP and its receptors change. For instance, in psoriatic plaques P2X_7_ receptors were found to be upregulated in the basal cell layer, suggesting that activation of KCs is facilitated by ATP (25). ATP is indeed elevated under pathological conditions, as it can be released by IFNγ activated and/or dying leukocytes and KCs (26, 27). Moreover, early results demonstrated defective hydrolysis of ATP in the psoriatic epidermis, leading to accumulation of extracellular ATP in the diseased skin, which supports the notion that ATP is profoundly involved in development of psoriasis (28). These early studies were recently confirmed by Killeen et al. (29), showing in the dermis of psoriatic lesions in a skin explant model elevated expression of P2X_7_ receptors as compared to healthy skin. This increased P2X_7_ signaling lead also to a phenotype of skin-DCs that predominantly induced Th17 cells, which are the main drivers of psoriasis. Finally, the elevated ATP concentrations in skin can also activate neutrophils, which in conjunction with IL-23, form a local inflammatory circuit maintaining psoriasiform dermatitis in mice (30). Therefore, increased levels of ATP together with enhanced expression of ATP receptors seem to be involved in maintaining an inflammatory environment in psoriatic skin.

On the other hand counter regulatory mechanisms directly related to the degradation product of ATP, i.e., Ado, have been described too. For instance, chronically stimulated epidermal KCs have an altered expression pattern of different Ado receptor (AdoR) types, with the rather pro-proliferative acting A_2_A receptor upregulated and reduced expression of the inhibitory A_2_B receptor (31). These and other observations led to investigations that utilize topical application of AdoR agonists for the treatment of psoriasis. Indeed, engagement of the AdoR A_3_ leads to reduced production of IL-17 and IL-23 in KCs of psoriatic patients, inducing amelioration of the disease (32, 33). Therefore, several drugs acting as agonist for different types of AdoR are currently used in clinical trials of skin- and other inflammatory diseases (34, 35). But not only in inflammatory diseases ATP plays a role, it is also important for induction of acute inflammation in skin. Weber et al. have shown that skin DCs without functioning P2X_7_ receptors are unable to sensitize T cell responses, indicating a role for directed ATP release as mediator of innate immune reactions (16). At the same time it became clear that haptens only act as trigger for hypersensitivity reactions when they induce release of ATP. Therefore, even experimental attempts were made to predict the “allergic potential” of chemicals by their ability to induce ATP release in KC cultures (36).

### ATP as substrate for adenosine production

A major degradation product of ATP is Ado, which can be generated intracellularly as well as extracellularly. Ado derives from the dephosphorylation of ATP, catalyzed by different enzymes: the ectonucleoside triphosphate diphosphohydrolase 1 (CD39) and the ecto-5'-nucleotidase (CD73) (37, 38). Both enzymes act sequentially in degrading extracellular ATP to adenosine. In a first step CD39 converts ATP to adenosine-di-phosphate and adenosine-mono-phosphate. In a second step the action of CD73 clips off the last remaining phosphate group, producing Ado (39). Ado can be released by nucleoside transporters from the cytoplasm of cells (4), however, the extracellular degradation of ATP by CD39 and CD73 is thought to provide the major pathway for regulating extracellular Ado concentrations. Its degradation is accomplished by adenosine deaminase (ADA), which exists in intra- as well as extracellular forms (40, 41). Extracellular ADA can bind to CD26 (42). Thus, similar to ATP and ADP, Ado can be degraded to inosine by cell membrane bound enzymes. In summary, the regulated destruction of extracellular ATP to Ado by enzymatic digestions offers cells a possibility to shape the tissue environment from a pro-inflammatory (high concentrations of free ATP) to a rather immunosuppressive (elevated levels of Ado) ambiance (43). As DCs express CD39 and/or CD73 as well as AdoR, they actively participate in immune responses affected by Ado (Figure 1).

### Regulation of extracellular Ado and ATP concentrations

In light of the opposing functions of the two mutually transformable signaling molecules ATP (activating) and Ado (suppressing) on immune reactions, their temporal/spatial distribution in tissues or along the plasma membranes of cells is of importance. Cells will presumably integrate activating (ATP) and suppressive (Ado) signaling pathways rendering a “final” outcome. Therefore, the half live as well as the diffusion speed through tissues is a critical factor determining the effects of ATP/Ado signaling. Real “in tissue” data of the distribution of extracellular ATP or Ado, respectively, are hardly available. However, contents in body fluids or organ cultures can be measured. For instance, in dog as well as human plasma Ado is only stable for a few seconds (44), making it a “short range” molecule. This rapid degradation may be useful to prevent a generalized immune suppression and it further prevents Ado from reaching the central nervous system, where it acts as neurotransmitter (45) and elevated levels may therefore disturb nerve functions. Moreover, a short half-life makes Ado a more defined tool for cellular communication. Because only cells that harbor CD73 on their surfaces are able to produce sufficient amounts of Ado that then acts locally by engaging AdoR of adjacent cells. This mechanism may in particular of importance for tolerance induction, as Ado production by CD73 expressing DCs is required during the intimate DC:T cell priming process in order to render T cells tolerant (own unpublished results). Finally, to regulate Ado concentrations in relation to ATP not only the half-life is important, also the regulation of expression of the Ado producing ectoenzyme CD73 provides a means to fine tune the extracellular Ado content. During ischemic preconditioning expression of CD73 is induced within 30 min (46), greatly enhancing the extracellular Ado concentration in tissues and thereby overcoming the degradation by ADA.

For ATP biosensors are available (47) making is more feasible to monitor extracellular ATP content in cell culture settings. The reported half live of ATP varies from up 2–20 min depending on the organ and the methods used (48–51). Of note, in the immune system ATP actions are rather fast, as neutrophils show a burst of ATP release for only 5 s after being stimulated with fMLP (52). However, these data are once more obtained in *in vitro* culture systems, which differ from the *in situ* situation, but after all these data give an impression on the speed and range of ATP or Ado signaling. It provides evidence that Ado may not act “cytokine-like” with distribution via the blood stream and exerting action(s) in tissues far from its origin.

## Effects of Ado on DCs

### Expression of Ado receptors by DCs

**Figure 2:**
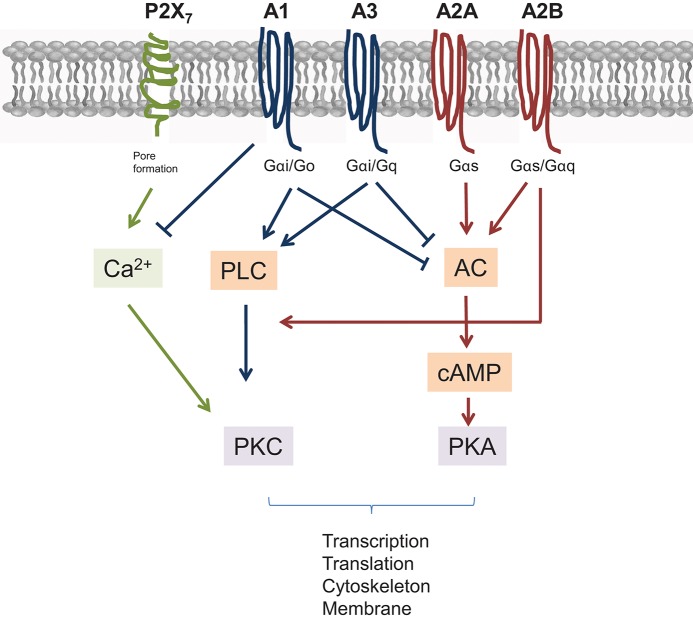
Scheme of major pathways and interconnections of AdoR and P2X_7_ ATP receptors. All receptors signal through G proteins. Different types of G proteins determine the further outcome. Briefly, A2A and A2B AdoR elevate adenylyl cyclase (AC) leading to activation of protein kinase A (PKA) through elevated cAMP levels. AC and thus cAMP is suppressed by A1 and A3 AdoR engagement, which themselves signal via phospholipase C (PLC) and proteinkinase C (PKC). However, raising Ca^2+^ levels, which transmit a P2X_7_ derived signal are blocked by A1 AdoR. Finally, A2B AdoR can augment signals derived from A1 and A3 as it stimulates Ca^2+^ mediated PKC activation also.

Four Ado Receptors (AdoR) are known so far (A_1_, A_2_A, A_2_B, and A_3_). Structurally they all belong to G-protein-coupled-receptors (GPCRs), but their intracellular signaling differs (Figure 2). In general the A_2_ receptor types are G_α*s*_-protein coupled receptors, with the A_2_B receptor additionally signaling via Gαq. In cells an activated G protein complex forms at the inner leaflet of the cell membrane after Ado engagement, which leads to activation of the adenylate cyclase (AC) and to rising cAMP levels (in case of G_α*s*_). As a consequence protein kinase A (PKA) is activated as secondary effector. On a molecular level this can directly be counteracted by engagement of A_1_ or A_3_ AdoR, which signal via G_α*i*_/G_α*q*_ complexes. Among them, the G_α*i*/*o*_ complex inhibits AC activity and thus dampens A_2_ mediated signaling. The main signal transduction of A1 and A3 receptors downstream of G proteins is mediated by phospholipase C induced secondary messengers that ultimately leads to increased Ca^2+^ levels and PKC activation. Thus, a different secondary effector is induced by A_1_ and A_3_ AdoR, resulting in activation of different sets of genes. But nevertheless, even here a crosstalk with the A_2_B receptors is possible, as A_2_B AdoR via its coupling to G_α*q*_ can feed into the PLC mediated pathway and support A_1_ and A_3_ AdoR signaling (53, 54).

Many reports show expression of all four subtypes of AdoR by DCs in varying degrees (55, 56). However, the levels of expression and their distribution among defined subset of DC remain uncertain. When analysing the available data on AdoR expression by DCs at a glance it becomes clear that AdoR expression correlates with the maturation status of DC. Human immature DC express A_1_ and A_3_ AdoR, which after engagement activate and recruit DCs to inflammatory sites (57). Upon maturation A_2_ AdoR emerge in DCs, now triggering rather inhibitory effects such as reduced secretion of IL-6, IL-12, and IFNγ (58). Here, differential expression of AdoR by DCs serves the purpose of regulating inflammatory processes. I.e., in the beginning of an insult, immature DCs are rendered active and are recruited to the inflammatory site whereas later A_2_-type AdoR expression limits over boarding inflammatory reactions. However, with several ways of cross talk between AdoR (as described above), differential expression by different cell types as well as varying affinities for purines, it is nearly impossible to assign one defined effect on cell physiology to the sole action of one AdoR or to one ligand *in vivo*. But *in vitro* studies can at least give insight into general pathways modulated by Ado.

### Effects of Ado on functions of DCs

Despite the fact that four different AdoR can activate different pathways at the same time that may have opposite effects on immune cell activation, many reports unequivocally demonstrate immune suppressive actions of Ado on DCs. In particular cAMP elevating AdoR A_2_A and A_2_B mediate rather inhibitory functions in DCs (53). For instance, after stimulation of respective AdoR *in vitro*, human DCs downmodulate secretion of IL-12 and TNFα. The cells expressed low amounts of MHC class II and were functionally impaired in stimulating proliferation of allogenic T cells. Further parameters of DC activation such as CXCL10, CCL2 and CCL12 secretion were also downregulated by Ado (56, 59–62). All of these features are indicators for a less mature phenotype of DC, which can be regarded as a tolerogenic type of DC (63).

In an even broader context a CD73^+^ cellular environment may be important to keep DC in “steady state” condition. *In vivo* genetic ablation of CD73 in mice leads to enhanced inflammatory reactions in a contact hypersensitivity model that is driven by increased migration of skin DCs to peripheral lymph nodes (64). Moreover, when analyzing the expression of T cell costimulatory molecules by different DC subsets after application of the hapten TNCB, we found increased expression of CD86 in subsets of skin DCs in CD73 deficient as compared to control mice. These data are further corroborated by findings using stimulation or blockade of Ado deaminase (ADA), an enzyme that is crucial for degradation of extracellular Ado. ADA is expressed by DCs during ongoing inflammation to degrade CD73 derived Ado and to maintain their hyper-reactive state (65). In contrast, in absence of ADA Ado levels in cellular environments are increased, as a consequence tolerogenic functions of DCs are enhanced (60). Moreover, addition of ADA to DC:T cell cultures, which leads to depletion of Ado from the cellular environment, enhanced priming of effector T cells and suppressed induction of Treg (66). In aggregate, adequate levels of extracellular Ado in peripheral tissues may be of importance to prevent overshooting DC activity and to maintain their “steady state,” which has been shown to be crucial for the tolerogenic function of DCs (67).

But beyond the mere prevention of DC maturation by Ado, the DC phenotype may be impacted in more fundamental ways. For example engagement of A_2_ AdoR in DCs enables them to actively suppress immune reactions. The mechanisms include the stimulation of IL-10 secretion or the upregulation of T cell inhibitory molecules such as B7H1, resulting in tolerant T cells as their proper activation by DCs is impaired (62, 68, 69).

Even “imprinting” tolerogenic functions in DCs has been attributed to AdoR engagement. Li et al. (70) were able to attenuate acute kidney injury by infusing DCs pretreated *ex vivo* with A_2_A AdoR agonists. This phenotype of Ado tolerogenic DCs was stable for more than a week and its action *in vivo* relies on impeding NKT cell activation by a so far unknown mechanism. AdoR expression can also be intrinsically upregulated by already immunosuppressive DC subtypes to bolster their immunoregulatory functions. For instance, in a tolerogenic pediatric DC subtype, IL-10 is upregulated after Fc receptor mediated stimulation along with increased expression of the A_2_A AdoR, which after Engagement further augments their IL-10 production (71). Thus, A_2_A AdoR expression helps to reinforce the immunosuppressive capacity of the DCs.

## Signaling of adoR in DCs

### The molecular mechanisms of camp in DCs

The main intracellular suppressive pathways triggered by A_2_ AdoR types involve cAMP as a second messenger. Roughly, both A_2_-type AdoR elevate cAMP levels by activating Adenylyl Cylase (AC). Further downstream cAMP signals via PKA that regulates gene transcription via NF-κB, HIF-1α and CREB. In addition, A_2_B AdoR also acts on PLC, inducing raising intracellular Ca^2+^ levels.

**Figure 3:**
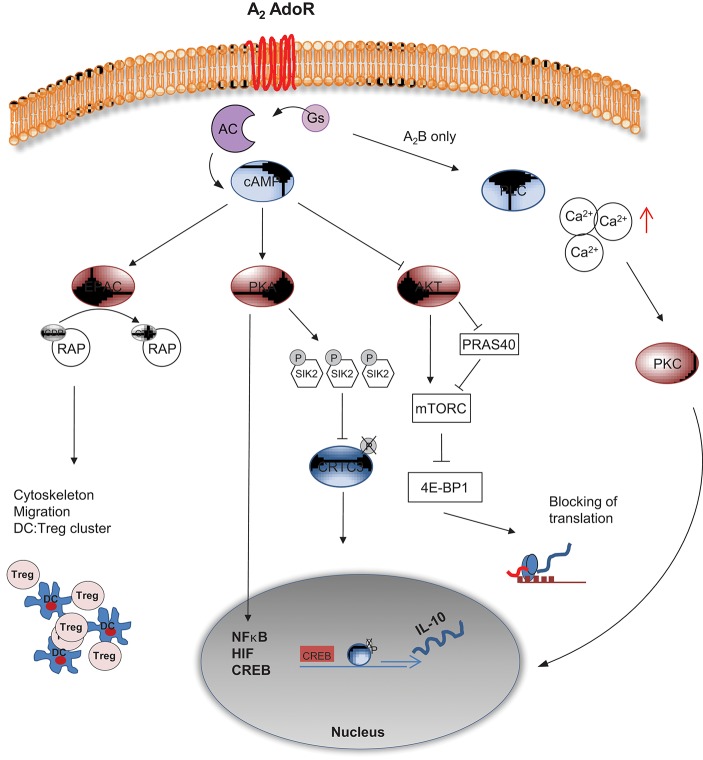
cAMP mediated intracellular signaling of A2 AdoR. A2A and A2B AdoR activate adenylyl cyclase and elevate cAMP levels. The A2B AdoR can concomitantly activate Phospholipase C (PLC), thereby triggering elevated levels of free Calcium (Ca^2+^). PKC mediated activation of NF-κB related gene expression follows. cAMP can suppress AKT activation leading to reduced mTORC activity either directly or via PRAS40. Without proper mTORC activity 4E-BP1 complex will terminate translation of proteins widely necessary for activation of cells. Additionally cAMP can signal via PKA, leading to hyper phosphorylation of Salt induced kinase (SIK) 2, allowing the phosphorylated transcription factor CRTC3 to cluster with CREB and to initiate translation of IL-10. Besides, further interaction of PKA with transcription factors such as NF-κB, HIF and CREB can induce “inhibitory” gene expression. A PKA independent pathway is mediated by EPAC, an enzyme that activates RAP via GTP binding, leading to profound changes in cytoskeleton and migration of DCs. As a result inhibitory DC:Treg clusters are formed and the immunological synapse may be changed in a tolerogenic fashion.

In a recent transcriptomic approach performed in bone marrow derived DCs (72), elevated activity of AC was connected to both, inhibition of AKT signaling and to activation of PKA (Figure 3). PKA has relevance for host defense capacities, as inhibition of Salt induced kinases (SIK) by cAMP-activated and PKA-mediated phosphorylation was shown to suppress secretion of the pro-inflammatory cytokines IL-6, IL-12, and TNFα by DCs and macrophages (73–75). Moreover, one of the SIK targets is the CREB-regulated transcription co-activator 3 (CRTC3) that can be phosphorylated at several serine residues. Phosphorylation of CRTC3 is inhibited by the cAMP-activated PKA, leading to translocation of the non-phosphorylated CRTC3 into the nucleus, where interaction with activated CREB upregulate IL-10 gene transcription (74).

In parallel to PKA activation, AKT activity is downregulated by elevated cAMP levels, promoting mTOR inhibition via PRAS40 (76). As a result, the downstream effectors of mTOR involved in the synthesis of cellular proteins, such as 4E-BP1 are hypophosphorylated. In this state, 4E-BP1 forms complexes with eukaryotic translation initiation factors and prevents translation (77). mTOR signaling regulation by AdoR driven cAMP content in DC may act as an important regulator of the antibacterial inflammatory response in monocytes, macrophages and primary dendritic cells (78, 79).

### Effects of adoR triggered camp levels on phenotype and function

Despite that AdoR triggered cAMP elevation has multiple molecular targets the overall effect is obvious, as several reports show clear induction of an immunocompromised and tolerogenic phenotype of DC by cAMP. This is indicated by reduced secretion of proinflammatory cytokines, reduced expression of MHC class-II but elevated secretion of IL-10. Also the capacity of DCs to prime CD8^+^ T cells *in vitro* was impaired in DCs with elevated intracellular levels of cAMP after induction by Ado or defined AdoR agonists such as 5′-N-Ethylcarboxamidoadenosine (62, 68, 80,–83). In turn, cAMP can feed back on AdoR expression. For example, high levels of cAMP induced by agents that trigger Gs-protein coupled receptors, upregulates expression of A_2_ AdoR in PC12 tumor cells (84). This cycle may therefore vigorously enhance Ado mediated suppressive effects in cells, as cAMP triggered upregulation of AdoR provides a means that leads to an even more sustained cAMP production.

To further delineate the possible cAMP effects that are mediated by AdoR engagement, one can artificially raise the cAMP content in DCs with Cholera toxin to mimic A_2_ AdoR triggering. This leads yet to another subtype of tolerogenic DCs, i.e., DCs that express both isoforms of the tolerogenic molecule cytotoxic T lymphocyte antigen 2 (CTLA-2α and CTLA-2β) (85). These DCs resembled a semi-mature state and were able to promote TGFβ-dependent Foxp3^+^ “induced” Treg conversion. Of note expression of CTLA-2 was critical for this function as genetic downregulation by siRNA reduced Treg conversion, while addition of recombinant CTLA-2α increased Treg conversion *in vitro*. Finally, when Lee et al. (81) investigated the role of DCs in priming of Th2 cells, they showed that deletion of genes that encode the GTP binding protein Gαs, leads to decreased cAMP signaling in DCs and provokes Th2 T cells with a prominent allergic phenotype. In contrast, increases in cAMP levels inhibited these responses. These findings imply that G protein-coupled receptors in DCs, such as A_2_ AdoR, which are natural regulators of cAMP formation, can prevent Th2-mediated immunopathologies by rendering DCs unable to induce potent Th2 answers.

Another major pathway induced by rising cAMP levels, but independent from PKA, depends on the exchange protein EPAC. Upon cAMP mediated activation, EPAC catalyzes the GTP binding of RAP1, a major regulator of the cytoskeleton. Via this axis cAMP seems to affect cell motility, cell adhesion, chemotaxis and phagocytosis (86). For DCs in particular it has been shown that Ado released by Treg is responsible for attracting them (mediated by an EPAC-RAP dependent pathway), leading to formation of DC:Treg aggregates (87). In these aggregates DC undergo “tolerogenic instruction,” as they start to produce IL-10, upregulate T cell inhibitory molecules and simultaneously downregulate expression of MHC class II molecules. Moreover, even the directed induction of DC:Treg clusters themselves may serve immunosuppressive functions, as Onishi et al. (88) have shown that Treg insolate effector T cells from proper activation by DCs by simply outcompeting them and keeping DCs in clusters.

### Priming of T cells by DCs in presence of Ado is altered

Despite the many well documented and long lasting effects of AdoR engagement on function of isolated DCs, the immediate presence of Ado during initial DC:T cell contact is crucially affecting the resulting immune response. For instance, *in vitro* engagement of A_2_A AdoR during the cognate MHC:peptide (as presented by DCs) T cell interaction leads to induction of T cell anergy and not to activation of T cells that normally ensues after DC:T cell interaction (89). This effect seems to be dependent on altered signaling in T cells, as reduced activation of the MAPK pathway was observed under these conditions. Ado:DC induced anergic T cells are not only refractory to restimulation, they also develop a CD25^−^ LAG3^+^ “regulatory” phenotype that actively prevents autoimmunity. Thus, the initial tolerogenic effects of Ado during antigen presentation by DCs will further be disseminated into tissues by these induced “regulatory” T cells.

As DCs can express CD73 themselves, production of extracellular Ado by DCs is conceivable and regulated expression of CD73 by DC subsets may one way to tune DC function for either tolerance (high CD73) or immunity (low CD73). Indeed, in a skin model for contact hypersensitivity application of the tolerogen 2,4-dinitrothiocyanobenzene (DNTB) rendered mice tolerant toward sensitization with the hapten 2,4-dinitrofluorobenzene (DNFB) (90). We found that induction of tolerance with DNTB was accompanied by increased expression of CD73 by skin migrating DCs and of note, in CD73 deficient animals tolerance induction by DNTB ceased (unpublished data). This underlines the importance of tissue derived Ado in governing DC functions under inflammatory conditions.

### The complex regulation of ATP–Ado signaling during inflammation

As DCs can express all four AdoR, the ectonucleotidases CD39/CD73 as well as P2X_7_ receptors, disentangling the ATP and Ado effects is very complex (91). It becomes even more complicated, as the different receptors transmit either stimulatory or suppressive signals, differ in their affinity for the respective ligands and are expressed to different degrees. The well investigated example of ATP induced chemotaxis of neutrophils gives an example how important the actual physical distribution of the different receptor molecules within a cell membrane is for their function. In neutrophils the chemotactic signal induced by fMLP is translated into ATP release by panx1. It will autocrinely act back on P2Y_2_ receptors. At the same time stimulatory A_3_ AdoR as well as CD39/CD73 are recruited to this part of the membrane, creating a local excitation circuit by activating PIP3, MAPK pathways and forming a “leading edge” for migration. A_2_A AdoR are excluded from this membrane site and are accumulating at the “trailing edge.” At the same time Ado, produced at the “leading edge” by activity of CD39/CD73, diffuses to the “back” of the cell and engages A2A receptors. This signal is transmitted by means of cAMP–PKA activation and suppresses the activation of the cell locally. As a result neutrophils are polarized and find their way along chemotactic gradients (92–94). Altogether this was an elaborative effort of several research groups and similar investigation can be done for DCs too. Here we are just at the beginning, just investigating broad effects of ATP/Ado on DC migration and DC activation, without knowing how the individual pathways are interconnected at a molecular level.

**Figure 4:**
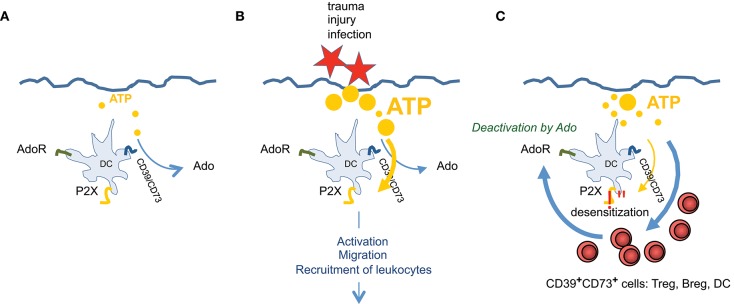
ATP and Ado are involved in regulating tissue inflammation. Exemplary for skin it is shown that **(A)** during steady state only trace amounts of ATP, derived from cellular detritus, are present. ATP will be degraded immediately by CD39/CD73^high^ Langerhans cells. **(B)** After injury high amounts of ATP are set free, which cannot be degraded effectively and overrule Ado production. This stimulates DCs via P2X_7_ receptors, leading to activation, to migration and to recruitment of different subsets of leukocytes. **(C)** When infection goes on, more CD39/CD73 expressing leukocytes are entering the tissue and P2X_7_ signaling ceases due to receptor desensitization and by counteraction of Ado. ATP is degraded to Ado which now prevails and terminates effector functions of various immune cells. Thus, immune homeostasis is reestablished.

Nevertheless, in a simplified scheme one can consider ATP as rather stimulatory and proinflammatory, and Ado (A_2_A and A_2_B receptors elevating cAMP) as being immune suppressive. In that sense CD39/CD73 expressing DCs are key cell for modulating homeostasis and inflammation and both receptor types (for ATP and Ado) are required to actually “measure” the degree of immune suppression or activation, respectively (Figure 4). Under non inflammatory conditions “steady state” DCs are patrolling through different tissues (95, 96) and sense only trace amounts of ATP, as tissues are intact and only limited amounts of extracellular ATP are produced, for instance by apoptotic cells. To maintain this homoeostatic status, high expression of CD39/CD73 ensures efficient degradation of ATP, preventing activation of the immune system. Examples are Langerhans cells in the epidermis that are highly positive for CD39 and degrade ATP effectively (37). Only when infection, tumor growth or trauma lead to elevated levels of extracellular ATP, the activating properties prevail, despite the fact that Ado receptors are expressed also. ATP simply outnumbers Ado effects. Subsets of immature peripheral DCs are recruited by ATP (58, 97) and an immune response is initiated. But counter regulatory mechanisms are initiated at the same time. For instance P2X_7_ receptors become refractory to repeated stimulation by high ATP concentrations (37), making the DCs insensible to ATP mediated activation (58). Moreover, recruitment of regulatory T cells to inflammatory sites, which express high levels of CD39 and CD73, accelerates the degradation of ATP to Ado (98, 99). So the balance tips toward an Ado enriched ambiance that progressively exerts anti-inflammatory functions. More Ado means reduced proinflammatory functions of DCs (69, 70, 81, 100, 101), less migration of DCs from tissue to lymph nodes (64) and increased induction of regulatory T cells (63, 60, 87, 89). Thus, slowly immune homeostasis is reestablished.

## Conclusion

The turnover of extracellular ATP to Ado by cell bound CD39 and CD73 offers a possibility to shape the tissue environment from an inflammatory (ATP high) to an immune suppressive habitat. DCs participate in this process as they (i) express ATP degrading enzymes CD39 and CD73 and (ii) harbor AdoR. Therefore, immunosuppressive effects of Ado can be mediated in two ways by DCs: First, DC derived Ado suppresses activation of T cells and fosters the induction of anergic and/or regulatory T cells during the cognate DC:T cell interactions. Secondly, Ado derived from adjacent cells act on DCs, preventing DC maturation and development of effector functions. These steady state DCs are considered tolerogenic. Thus, an Ado enriched tissue environment may be of importance to maintain the “steady state” of DCs to prevent autoimmunity.

## Author contributions

CS-V and SR contributed equally. SR prepared figures and CS-V performed experiments. All authors wrote the paper.

### Conflict of interest statement

The authors declare that the research was conducted in the absence of any commercial or financial relationships that could be construed as a potential conflict of interest.
